# Dam *Mycobacterium avium* subspecies *paratuberculosis* (MAP) infection status does not predetermine calves for future shedding when raised in a contaminated environment: a cohort study

**DOI:** 10.1186/s13567-015-0191-2

**Published:** 2015-06-19

**Authors:** Susanne WF Eisenberg, Victor PMG Rutten, Ad P Koets

**Affiliations:** Department of Farm Animal Health, Faculty of Veterinary Medicine, Utrecht University, Utrecht, The Netherlands; Division of Immunology, Department of Infectious Disease and Immunology, Faculty of Veterinary Medicine, Utrecht University, Utrecht, The Netherlands; Department of Veterinary Tropical Diseases, Faculty of Veterinary Science, University of Pretoria, Pretoria, South Africa; Central Veterinary Institute, Wageningen University and Research Centre (CVI-Lelystad), Wageningen, The Netherlands

## Abstract

Uptake of *Mycobacterium avium* subsp*. paratuberculosis* (MAP) by calves in the first days of life from colostrum, milk and faeces is regarded an important moment of transmission. The objective of this study was to quantify the association between the MAP status of dams as determined by the presence of MAP DNA and antibody in colostrum and that of DNA in faeces and the environment with subsequent MAP shedding of their daughters. A cohort of 117 dam-daughter pairs giving birth/being born on eight commercial dairy farms with endemic paratuberculosis was followed where colostrum, faecal and environmental samples (dust) were analysed for the presence of MAP using an IS900 real-time PCR. Antibodies in colostrum were measured by ELISA. Analysis of dust samples showed that on all farms environmental MAP exposure occurred continuously. In significantly more colostrum samples (48%) MAP DNA was detected compared to faecal samples (37%). MAP specific antibodies were present in 34% of the colostrum samples. In total MAP DNA was present in faecal samples of 41% of the daughters at least once during the sampling period. The association between faecal shedding in the offspring and the dam MAP status defined by MAP PCR on colostrum, MAP PCR on faeces or ELISA on colostrum was determined by an exact cox regression analysis for discrete data. The model indicated that the hazard for faecal shedding in daughters born to MAP positive dams was not significantly different compared to daughters born to MAP negative dams. When born to a dam with DNA positive faeces the HR was 1.05 (CI 0.6; 1.8) and with DNA positive colostrum the HR was 1.17 (CI 0.6; 2.3). When dam status was defined by a combination of both PCR outcomes (faeces and colostrum) and the ELISA outcome the HR was 1.26 (CI 0.9; 1.9). Therefore, this study indicates that neither the presence of MAP DNA in colostrum, MAP DNA in faeces nor the presence of MAP antibodies in colostrum of the dam significantly influences the hazard of MAP shedding in their subsequent daughters up to the age of two years when raised in a contaminated environment.

## Introduction

*Mycobacterium avium* subspecies *paratuberculosis* (MAP) is the causative agent of paratuberculosis or Johne’s disease in cattle. The disease is characterized by chronic diarrhoea, weight loss and loss in milk production [1]. It occurs in countries worldwide with herd prevalence’s in Europe and the US of over 50% [2,3]. Therefore, it has substantial economic impact on the dairy industry [4].

It is generally accepted that uptake of MAP by calves in the first days of life is most important for MAP transmission [1,5]. After infection the disease may be in a subclinical phase lasting 2–5 years, whereas only some animals develop clinical disease. Infected dairy cattle shed MAP intermittently in their faeces with increasing amounts when clinical disease develops [1]. Shedding in milk and colostrum has been described [6,7] although not much information regarding the use of colostrum as a substrate for MAP diagnostics is present [8]. Calves born to MAP positive dams have a higher infection risk due to the possibility of in-utero transmission [9] but also due to contact with infectious faeces and uptake of infectious colostrum at parturition [10-12]. Therefore, control programs are not only built on removing test-positive cows but do also emphasize calf hygiene measures such as separation of dam, calf directly after parturition and feeding colostrum of a single MAP negative dam and removal of calves born to MAP positive cows [13-16]. However, paired dam-daughter data supporting the general opinion of the higher MAP infection risk of calves born to MAP positive dams is scarce. In addition, due to the long incubation period of disease and the low sensitivity of available tests in early disease it is hard to differentiate between pre- and postnatal transmission. Two studies described a decrease in infection risk when feeding colostrum-replacer or pasteurized colostrum to calves implying that raw colostrum could be considered a source of MAP infection [11,12]. In contrast, two studies that followed-up calves after MAP exposure which was defined as “dam faecal culture positive” or “administered PCR positive colostrum” respectively did not reveal an increased infection risk for calves born to MAP positive dams [17,18]. However, a retrospective study identified a higher risk for cows to be ELISA positive when their dams were ELISA positive as well [10].

The main objective of this study was to investigate the association between dam MAP status, and subsequent shedding of MAP of daughters born to these dams without differentiating between pre- and postnatal transmission. Three risk factors were examined in this study. The MAP status of the dam determined by immunological and microbiological diagnostic assays at calving. As second risk factor the quality of dam’s colostrum as administered to the calves during the first 24 h of life was evaluated. Colostrum quality was defined by the quantity of immunoglobulins, the presence of MAP specific immunoglobulins and the presence of MAP DNA. Apart from the MAP specific aspects of colostrum quality, the quantity of immunoglobulins in colostrum is an important factor in calf growth and morbidity during rearing [19] and quality differences may therefore be a potential confounder in determining the risk of MAP infection. As a third risk factor MAP exposure around calving and during the follow-up period was monitored using environmental sampling.

## Materials and methods

### Study design

In this cohort study eight farms with a known MAP history defined by either the Intensive Paratuberculosis Program (*n* = 3) or the Bulk Milk Quality Assurance Program (BMQAP, *n* = 5) were enrolled [20,21]. Farms were situated in Friesland, a province in the north of the Netherlands. The initial within-farm paratuberculosis prevalence was estimated by MAP milk ELISA in the first month of the study. The study population consisted of all dams giving birth to female calves between October 2009 and March 2010 on these farms and their heifer calves. Farmers were instructed to sample colostrum as it was administered to the calf and to collect rectal faecal samples of dams at parturition using sterile sampling materials supplied. Samples were stored at −20 °C until transport to the Faculty of Veterinary Medicine (Utrecht, The Netherlands) at the next farm visit with a maximum time to collection of four weeks. Information regarding age at birth and parity was collected as well. In autumn 2011 and 2012 at approximately one and two years of age faecal samples of all enrolled heifer calves still present on the farms were collected. Additionally, these farms were enrolled in an intensive MAP surveillance scheme and were visited every 4–6 weeks for a 2-year period. During that time, environmental dust samples as a proxy for environmental MAP exposure were collected.

### Animals

Cows were separated from the dry cow herd several days before the expected parturition into a calving pen where cows were housed individually. Farmers checked for giving birth at least 4 times a day. The calf was separated shortly after parturition with a maximum time span of six hours. Before separation colostrum was collected to feed to the calf. The pen was cleaned in between calvings by removing faecal contaminated bedding material. Calves were housed in individual pens for the first two weeks of life. Subsequently they were transferred into group pens with age differences per group of three months maximum. Dust samples were collected in the all dairy and young stock barns. Detailed information about dust sampling, farm layout and basic farm characteristics has been published previously [22,23]. In short, dust samples were collected using electrostatic dust collectors (EDC) that were replaced at every farm visit. Electrostatic microfiber wipes (Zeeman, Alphen a/d Rijn, The Netherlands) were used as EDC and at each farm six dust collectors were installed at fixed locations in dairy and young stock housings. Sampling locations were two meters above the floor to assure only settled dust was collected, cows could not reach them and therefor destruction was prevented and interference with routine management actions of the farmers was avoided. Environmental dust samples, colostrum and faecal samples were subsequently stored at −20 °C until further analysis.

Dam MAP status was defined by the outcome of the colostrum PCR (“PCR_col_”), the outcome of the colostrum ELISA (“AB_col_”) and the outcome of the faeces PCR (“PCR_faeces_”). Environmental MAP exposure was estimated by the number of MAP positive EDC’s collected during the whole study period. Faecal shedding of calves was established when MAP DNA was detected by the IS900 PCR in at least one of the two faecal samples collected (status 1), all others were marked as MAP test negative (status 0).

### Determination of [Immunoglobulin] of colostrum

Samples were centrifuged at 2500 *g* (Beckman Allegra X12-R Centrifuge) for 10 min to remove the milk fat content. Commercial Bovine Ig quantitation kits were used (Bethyl Laboratories, Inc, USA) according to instructions provided by the manufacturer. In short, samples were diluted 1:300 000 for IgG and IgG1 analysis and 1:30 000 for IgG2, IgM and IgA analysis after testing dilution rows. Quantitative determination of the immunoglobulin concentrations were performed for IgG heavy chain, IgG1, IgG2, IgA and IgM. The ELISAs were performed in duplicate in 96-wells plates (Costar 9018, Certified high binding-96 wells plate), with a duplicate reference row on each plate. Differences between duplicates of <10% were accepted and ELISA backgrounds were subtracted from the absorbance. Non-specific binding to the 96-wells plate was blocked using a commercially available blocking reagent (“Blocking reagent for ELISA”, Roche Diagnostics GmbH, Germany). Horseradish peroxidase (HRP) conjugated antibodies were used as capture antibodies and tetramethylbenzidine (TMB) for substrate and H_2_SO_4_ for stopping reaction. Absorbance was measured with a Thermo Scientific Multiscan FC at 450 nm. The reference plot was performed by Log-transforming of the reference concentration as well as the absorbance. For accurately converting sample absorbance to immunoglobulin concentration, a linear reference plot with a R2 > 0.98 was performed for each plate.

### ELISA on colostrum and milk samples

Colostrum and milk samples were analysed for the presence of MAP antibodies using the Pourquier ELISA (IDEXX Europe B.V., Hoofddorp, The Netherlands) according to the manufacturers manual. To determine if colostrum and milk samples were ELISA-positive, a sample-to-positive (S/P) ratio of 40 was used as a cut-off value as recommended by the manufacturer.

### MAP IS900 real-time PCR on faeces and colostrum

Nucleic acid present in colostrum and faecal samples was isolated using the MagMAX™ Total Nucleic Acid Isolation Kit (AmbionH MagMAX™ Total Nucleic Acid Isolation Kit, Applied Biosystems, Foster City, CA, USA) according to manufacturer’s instruction. For nucleic acid extraction 0.3 gram of faeces and 0.3 mL of colostrum were used. Isolated nucleic acid was screened for the presence of MAP DNA using an IS900 real-time PCR with a program with 45 cycles using a probe described previously [24].

### MAP presence in dust samples

The EDC were treated for dust preparation as described before and MAP detection was performed using a liquid culture technique, according to the protocol for para-JEM automated MAP culturing provided by TREK Diagnostic Systems (Cleveland, OH, USA) [23]. In short, all samples were incubated for 42 days regardless of a positive signal before day 42 and were subsequently tested by IS900 real-time PCR with a program with 45 cycles to confirm the presence of MAP DNA. Samples were designated “viable MAP positive” if the PCR showed a positive signal. The results of the EDC analysis were expressed dichotomously (1 = viable MAP positive; 0 = viable MAP negative).

### Statistical analysis

Descriptive statistics, summarizing test results and animal characteristics as well as data analysis were performed using IBM SPSS 20 (IBM Corp., Armonk, NY, USA).

Outcomes of PCR_col_ and PCR_faeces_ and loss of follow-up between exposed and unexposed daughters were compared by χ2 statistics. Ig concentrations between MAP IS900 PCR positive and negative colostrum samples were compared using a Students *t*-test. Numbers of positive EDC’s per farm were compared with one way ANOVA. To investigate the association between MAP test positivity of daughters up to the age of 2 years and the point MAP exposure by their dams at parturition an exact cox regression model for discrete data was performed using R statistical software (version 3.1.2, R Foundation for Statistical Computing, Vienna, Austria). The following equation was fitted: H(t) = H_0_(t)x exp(b_1_X_1_ + b_2_X_2_ + υ _1_ ) where H_0_(t) is the baseline hazard for heifers to become MAP shedders, b_1_ is the coefficient for MAP exposure, b_2_ is the coefficient for “parity” and υ_1_ represents the log-frailty of factor herd. In different runs of the model three MAP exposure variables were studied being exposure to PCR positive colostrum (Yes/No), to PCR positive faeces (Yes/No) and a combination of both PCR outcomes with the ELISA result (Yes/No). Confidence intervals (CI) of HR are presented as 95% CI. For the combined test result a dam was considered negative when all tests were negative, in all other cases the dam was classified positive (parallel interpretation of test results). Differences with *p* < 0.05 were considered statistically significant.

## Results

### Farms

Farms enrolled had between 79 and 137 cows (median 84) during the first study month. The rolling year average of milk production of the herds varied between 7028–10 354 kg/cow (median 8869). The estimated within-farm MAP prevalence based on milk ELISA outcome at that time varied between 0 and 16% (median 4.5%). During the 6 month intake period a total of 129 heifer calves were born. In total 117 “dam- daughter” pairs were analysed. In the first year of the study 12 daughters were lost to follow-up (8 exposed/4 unexposed) and at the second follow-up sampling 9 animals were lost to follow-up (5 exposed/4 unexposed). No significant difference of loss to follow-up between exposed and unexposed daughters was identified. All lost to follow-up daughters died or were culled for various reasons unrelated to paratuberculosis according to the farmers statement.

### Dams

**Figure 1:**
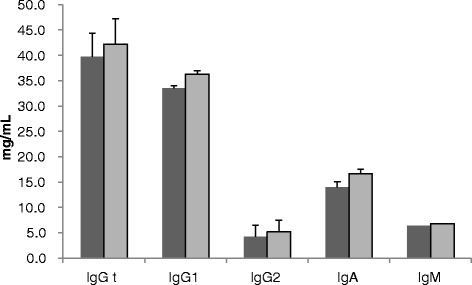
**Comparison of immunoglobulin concentration in colostrum between MAP IS900 PCR positive and MAP IS900 PCR negative colostrum.** Colostrum Immunoglobulin (Ig) isotype concentration of MAP IS900 positive (dark grey bars; *n* = 56) and negative (light grey bars; *n* = 61) colostrum samples in mg/mL + SEM. No significant difference in Ig isotype concentrations between both groups was detected for any of the isotypes studied.

None of the dams enrolled in the study showed any clinical signs of Johne’s disease such as diarrhoea, weight loss or oedema. MAP prevalence of the 117 enrolled dams determined by milk ELISA was 11%. Of all colostrum samples analysed 48% (56/117) were MAP IS900 PCR positive, whereas 34% (39/117) contained MAP specific antibodies (Table 1). Cows with IS900 PCR positive colostrum had similar quality colostrum in terms of total IgG concentration (IgGt) and IgG1, IgG2, IgA and IgM concentrations (Figure 1), compared to cows with IS900 PCR negative colostrum.

**Table 1:** **Descriptive statistics regarding dam characteristics at parturition enrolled in this study**

		**All tests negative**	**Colostrum**		**Faeces**	**EDC**	
Farm	*n*		ELISA	PCR	PCR	Collected	PCR
1	10	1	4	4	6	126	38
2	23	10	8	9	1	150	11
3	16	5	4	9	2	150	12
4	14	0	6	12	7	144	44
5	12	1	7	6	3	132	15
6	14	5	5	5	1	150	4
7	12	2	3	3	8	144	30
8	16	4	2	2	10	138	6
**Total**	**117**	**28**	**39**	56*	43*	**1134**	**160**
**%**		**24**	**34**	**48**	**37**		

Overview of status of dams which gave birth to a heifer- (calf) enrolled in this study per farm and the results of the *Mycobacterium avium* subspecies *paratuberculosis* (MAP) IS900 PCR on colostrum, faecal and dust samples, the results of the MAP ELISA on colostrum samples and the agreement of the test results.

*n: Number of dams.*

*p < 0.05.

Concurrent presence of MAP DNA and antibodies could only be detected in colostrum of 16% (19/117) of the dams. Presence of MAP DNA in faeces occurred in 37% (43/117) of the dams on the day of calving whereas 19% (22/117) of the dams had MAP IS900 PCR positive colostrum and faeces. Significantly more dams tested positive for MAP IS900 PCR in colostrum as compared to faecal matter (*p* = 0.032). Faecal PCR and colostrum ELISA were both positive in 11% (13/117) of the tested dams. Finally, in 3% (4/117) of the dams all three tests showed a positive result. Overall (all tests combined) 76% (89/117) of the dams were positive in one or more MAP specific diagnostic assays on the day of parturition. Consequently, 24% (28/117) of the dams was negative in all applied paratuberculosis diagnostic tests.

### Daughters

As outlined in detail in Table 2, of the 117 daughters enrolled in this study 66% (77/117) were exposed to MAP DNA at parturition, either through presence in faeces of the dam (18%; 21/117), through MAP IS900 positive colostrum (29%; 34/117) or both (19%; 22/117) (Table 2). Colostrum containing MAP antibodies was administered to 33% (39/117) of the heifer calves. In 15% (18/117) of the cases calves were fed colostrum which was MAP antibody and DNA positive whereas 18% (21/117) of the calves were fed colostrum which contained only MAP antibodies. Four animals (3%) were fed MAP DNA and antibodies in colostrum and were potentially exposed to MAP through IS900 PCR positive faeces at the same time. MAP IS900 PCR positive colostrum was administered to 48% (56/117) of the calves.

**Table 2:** **Descriptive statistics heifer (calves) exposure to MAP at parturition**

**MAP status dam at parturition**				**MAP status calves**	
**Colostrum PCR**	**Colostrum ELISA**	**Faecal PCR**	**N**	**Positive N**	**% positive**
+	-	-	20	5	25%
+	+	+	4	3	75%
+	-	+	18	5	28%
+	+	-	14	4	28%
-	+	-	12	5	42%
-	-	+	12	0	0%
-	+	+	9	3	34%
-	-	-	28	10	36%

Exposure of heifer- (calves) to MAP, as assessed by the dams infection status during parturition, and their cumulative infection status determined by MAP PCR of faeces at 1 and 2 years of age. -: test negative; +: test positive.

Of the 56 calves which received MAP IS900 PCR positive colostrum 32% (18/56) tested positive for MAP after the 2 year follow-up. After 2 years follow-up 35 daughters were identified as MAP positive by MAP shedding. MAP positive heifers were born to dams in all MAP test combinations varying from 36% (10/28) positive calves born to dams negative in all three tests up to 75% (3/4) positive calves born to dams with positive outcomes in all three tests.

### Environmental dust samples

During the two year period dust was sampled at 23 (SD2) farm visits. During the whole study period on average 141 (SD 9) dust samples were collected per farm. MAP positive EDC’s per monthly farm visit varied between 0–5 (out of 6). Summarized over the whole sampling period MAP positive EDC’s per farm varied between 4 and 44. Total numbers of MAP DNA positive dust samples collected per farm over the 2 year period varied significantly from each other (*p* < 0.05). More detailed information about EDC outcome has been published previously [22].

### Risk factors to become a MAP positive heifer

The cox regression analysis with either of the three exposure definitions did not identify a significant association between MAP exposure and the hazard of MAP DNA positive faeces later-on in life. The exposure to MAP DNA in colostrum revealed a HR 1.17 (CI 0.6; 2.3), the exposure to MAP DNA of faeces a HR of 1.05 (CI 0.6; 1.8) and the HR for the exposure to MAP DNA in faeces and colostrum and MAP antibodies was 1.26 (CI 0.9; 1.9) compared to the unexposed daughters. Therefore, none of the determined risk factors did increase the hazard for MAP shedding significantly. Since the proportional hazard assumption was not violated the difference in risk of MAP infection between “exposed” and “unexposed” did not change over time. Environmental exposure defined by the presence of MAP positive EDC’s occurred on all farms throughout the study period.

## Discussion

Postnatal transmission of MAP to susceptible calves has been described to occur mainly during the first year of life with the highest risk when exposure occurs within the first 6 months [1,25]. The first days of life may stand out in risk even more through high susceptibility of new born calves and a high likelihood of contact with infectious material during birth, in the calving environment and colostrum uptake [4,5]. However, differentiating between pre- and postnatal exposure is difficult. Our current study indicates that the MAP status of the dam does not predetermine calves for future MAP shedding when these calves are raised in a MAP contaminated environment.

The different diagnostic tests used in this study to determine MAP status of the dam agreed poorly since only 4 dams were found MAP positive by all three tests. The number of colostrum samples containing MAP DNA was significantly higher compared to the faecal shedding or presence of antibodies. Low test agreement when comparing different diagnostic MAP tests has been reported in literature before; however, these studies compared milk with serum ELISA, milk with faecal culture or milk, blood and faecal PCR [26-28]. Colostrum is not commonly used for the assessment of MAP infection, therefore only little information is available regarding the prevalence of antibody or antigen positive colostrum samples in commercial dairy herds. One recent study found a 130 times higher odds for dairy cows to have MAP antibody positive colostrum at 0 DIM compared to 4 DIM indicating that colostrum would have enhanced diagnostic value [8]. Our current results support these observations as the herd prevalence of MAP was a lot higher when determined by colostrum ELISA compared to milk ELISA. Previous studies on the presence of MAP organisms or DNA in faeces and colostrum indicated a higher number of cows shedding MAP in their faeces compared to MAP DNA presence in colostrum [6,29]. Cows that were heavy faecal shedders were more likely to shed the organism in colostrum than were light decal shedders [6]. In contrast, we identified a significant higher number of cows with MAP DNA positive colostrum compared to faeces. Whether the presence of MAP DNA in colostrum indicates actual shedding or environmental contamination during milking cannot be differentiated. MAP presence in faeces or in the environment signifies a higher risk of exposure without providing information as to actual uptake. In contrast, it is more likely that when MAP DNA positive colostrum was fed to the calf by the farmer during the first day of life MAP has been taken up by the calf.

In earlier research MAP transmission associated with MAP status of the dam was only assessed by one diagnostic test which determined either faecal shedding of MAP by the dam at day of birth, presence of MAP in administered colostrum or positivity for MAP specific antibodies of the dam [17,18]. These studies failed to identify dam status as a major risk factor for MAP transmission. Other studies reporting an increased risk of transmission by MAP positive dams used serology to classify the dam and enrolled significantly higher numbers of cows [10,30]. These data were often retrospective and failed to pinpoint exposure to a specific moment and could therefore represent an overall measure of environmental exposure instead combined with prenatal transmission. Due to the long incubation period the length of follow-up of the daughters has been identified to be critical [31]. In addition, herd prevalence was shown to be an important confounder indicating that only in low prevalent herds dam status is an important risk factor probably because in high prevalent herds the contamination of the environment is more important for transmission than dam status [31,32]. The current study measured three dam dependent indicators of dam MAP status as point MAP exposure at calving and its relation with MAP transmission to susceptible calves.

One weakness of the study was that due to low sensitivity of available diagnostic tests for determining disease status in individual cows in early disease, misclassification of dam status in the “all test negative” group might have occurred. However, since the objective of the study was investigating if point exposure of the calf at parturition could be identified as a risk factor and these dams were confirmed as not shedding MAP neither in colostrum nor in faeces at parturition the potential misclassification does not interfere with the research question. This non-differential misclassification of “dam status” would bias the HR towards null. Intra-uterine transmission of MAP has been described to occur in clinical but also in lower levels in subclinical infected cows and was not controlled for in this study [33,34]. Another limitation of the study is the long study period. Even when exposure at parturition was determined it is difficult to differentiate between infection due to exposure to MAP at parturition and infection due to exposure during calf rearing. But due to disease characteristics and diagnostic methods available long follow-up periods are necessary to be able to identify the outcome. It is often stated that calves are most susceptible in the first days of life and transmission occurs most effectively from dam to calf at parturition. However, recent data showed that a clear age dependent susceptibility does not exist because calves up to one year of age were successfully infected with MAP under experimental conditions [25]. But if transmission in the first day of life is most effective the difference between exposed and unexposed daughters is not expected to diminish during calf rearing.

Daughters in this study were followed up to only 2 years of age although MAP is known for its long incubation period [1]. Nevertheless, during this period the animals were sampled twice and 30% were identified as shedding MAP DNA positive faeces at least once. That faecal positive animals tested negative in subsequent tests is consistent with literature when a peak of MAP shedding in young stock was identified between 7–14 months with a decline afterwards [35]. In addition, diagnosis in early infection has been described as difficult since faecal shedding only occurs in low numbers or intermittently [36-38]. The onset of faecal shedding before the age of 2 years has been shown to be dependent on within-farm MAP prevalence with a maximum of 8 % of the young stock already shedding when MAP prevalence is below 20% as was the case in herds participating in this study (0-16% prevalence) [39]. However, the fact that one third of enrolled daughters were diagnosed as MAP positive at least once during the study indicates that MAP shedding in young stock does occur regularly. These findings are in agreement with recent literature that emphasizes the importance of young stock shedding when MAP control programs are incorporated on a farm [32,39,40]. The shedding of MAP in young stock can also be considered a risk factor for calf-to-calf transmission during young stock rearing although experimental studies have shown that calf-to-calf transmission occurs not frequently and modelling studies show the effect to be limited compared to the effect of the farm environment [41-43].

Daughters, MAP positive after 2 years, were born to dams with all combinations of test results, even from those negative in all performed tests at parturition, indicating that the identification of a predominant risk factor when calves are raised in an contaminated environment is difficult. The cox regression model did not identify a significant increase of the hazard to become MAP faeces PCR positive for daughters of MAP positive dams compared to daughters of MAP negative dams. Therefore, the model indicated that neither the uptake of MAP DNA positive colostrum nor the exposure to MAP DNA positive faeces at parturition could be identified as a statistically predominant risk factor. The combined exposure to MAP antibody and MAP positive colostrum which was shown to enhance MAP uptake by Peyer’s patches under experimental conditions did not increase the hazard of transmission either [44].

The distribution of cases as found in this study where a proportion of only 28% of MAP positive heifers was detected MAP exposed at parturition compared to 40% was MAP unexposed implicates an important role for the environment which has been published to be important before [10]. For example, the OR for a calf being born to a seropositive dam to become seropositive was estimated 3.6. However, when exposure to flush water was added to the model the OR for the effect of exposure to flush water was estimated to be 28.5 whereas dam status was only 6.6. and dam age at birth did not influence the outcome at all [10]. On the other hand, studies looking into the relation between feeding raw colostrum versus colostrum replacer or uptake of MAP positive versus MAP negative colostrum testing the hypothesis that MAP antigen in colostrum might increase MAP infection in exposed calves did not find significant differences [11,17]. Both studies stated that a post-hoc power-analysis indicated a lack of power to detect the desired effect with statistical significance. This might be also true for the study presented here where 48% of the calves were exposed to MAP DNA via positive colostrum. The percentage of calves with no potential exposure at parturition was relatively small (24%). In addition, all calves enrolled were potentially exposed to MAP DNA based on environmental testing which indicates a high level of MAP exposure when calves are born and raised in infected herds. Performing a post-hoc power analysis on data presented in this study revealed that when only the measure “MAP IS900 positive colostrum uptake” was used as exposure at birth an incidence difference between exposed and non-exposed of at least 26% would have been necessary to provide sufficient statistical power (>80%) to allow the detection of a difference between calves exposed and not-exposed during parturition at a significance level (α) ≤ 0.05. This analysis indicates that on farms with environmental MAP contamination exposure at parturition might not be as important for disease transmission as continuous MAP exposure through the environment which was confirmed by the presence of MAP in dust collected of all farms. The number of positive samples differed significantly between farms and therefore probably the level of exposure.

In conclusion, the results of this study indicated that the hazard for daughters which were MAP exposed at parturition and those which were unexposed to become MAP shedders did not significantly change. Results indicate that on farms with environmental MAP contamination exposure at parturition through MAP DNA containing colostrum or faeces might not be as important for disease transmission as continuous MAP exposure through the environment.
